# Posterior Reversible Encephalopathy Syndrome in a Patient with Variegate Porphyria: A Case Report

**DOI:** 10.7759/cureus.3351

**Published:** 2018-09-24

**Authors:** Faiza Rasheed, Qasim S Mehdi, Shoaib Bhatti, Muhammad Mannan Ali Khan

**Affiliations:** 1 Pediatrics, National Institute of Child Health, Karachi, PAK; 2 Internal Medicine, Ziauddin University, Karachi, PAK; 3 Orthopaedics, Dow University of Health Sciences, Karachi, PAK

**Keywords:** varigate porphyria, visual disturbances, hypertension, seizures, posterior reversible encephalo

## Abstract

Variegate porphyria (VP) is one of the groups of rare inherited disorders of hemoglobin synthesis called Porphyria. It has two distinct manifestations, that is, those of cutaneous and nervous system. Posterior reversible encephalopathy syndrome (PRES) is a rare complication of porphyria. It occurs due to vasogenic edema in white matter of predominantly parieto-occipital lobes, characterized by headache, visual disturbances, altered mental state, hypertension, and seizures. We report a child diagnosed with VP who presents with clinical signs and radiological manifestations suggestive of PRES. To our knowledge this has never been reported in a case of VP and only twice been reported in another type of porphyria.

A 12-year-old pre-pubertal boy already diagnosed with VP presents with seizure, visual disturbance, altered mental status, headache, and hypertension. Initial brain magnetic resonance imaging (MRI) revealed bilateral increased signal intensity in parieto-occipital region. Neurological opinion suggested that the symptoms experienced by the patient seem to be a complication of porphyria. Treatment was to control hypertension and prevent use of any aggravating agents. Follow-up MRI after two weeks revealed interval reduction in disease process. Diagnosis of PRES was thus confirmed.

PRES should be considered in patients presenting with symptoms typical of encephalitis/meningitis/acute disseminated encephalomyelitis in a patient suffering from porphyria. Early diagnosis is key to quick improvement and prevention of complications. Though rare in pre-pubertal patients, it should be kept as a possibility especially when patients present with hypertension. Care should be taken to not use any drugs that can trigger PRES.

## Introduction

Acute porphyria is a group containing four further types: Acute intermittent porphyria (AIP) (commonest), hereditary coproporphyria, variegate porphyria (VP), and aminolevulinic acid dehydratase porphyria (rarest). The former three are inherited in an autosomal dominant way resulting from deficiency of an enzyme essential in heme synthesis. VP is caused by a deficiency of the enzyme protoporphyrinogen oxidase (PPOX). VP usually presents in post-pubertal age with attacks of abdominal pain, skin manifestations, neurological as well as psychiatric disturbances [1,2]. Porphyrias in general are known to have many precipitating factors including certain medications (metabolized by the cytochrome P450 system), alcohol use, infections, low caloric intake, and changes in sex hormone balance during the menstrual cycle [1].

Posterior reversible encephalopathy syndrome (PRES) is a clinico-radiological entity and a rare complication of acute porphyria [3]. It occurs due to vasogenic edema in white matter of the brain predominantly affecting the posterior occipital and parietal lobes of the brain [4]. It is also known as hypertensive encephalopathy, a neurotoxic state that occurs secondary to the inability of posterior circulation to auto-regulate in response to acute changes in blood pressure. It is often but not always associated with hypertension [5,6]. PRES is characterized by headache, seizures, altered consciousness, and visual disturbances associated with potentially reversible neuro-radiological abnormalities [2]. If promptly recognized, the syndrome is easily reversible within a week [6,7]. PRES is an increasingly diagnosed disorder, but is still poorly understood [8]. We believe many cases of PRES are misdiagnosed due to lack of knowledge and this can result in potentially catastrophic outcomes.

Our case is a 12-year-old pre-pubertal male child, known case of VP, who presents with abdominal pain, loss of vision, altered level of consciousness, seizures, and acute hypertension suggestive of PRES. Magnetic resonance imaging (MRI) of brain correlates the diagnosis. While PRES has been reported with AIP only two times in international literature, it has never been reported with VP, making our case the first of its kind [2].

## Case presentation

We describe a 12-year-old child, diagnosed case of VP, who presented to the emergency department of National Institute of Child Health (NICH) on 25-11-2017 with abdominal pain for seven days, loss of vision and altered level of consciousness for two days, and seizures for one day. The patient was previously diagnosed as a case of variegate porphyria on May 2016 while he was admitted in Civil Hospital Karachi, when a urine sample sent to Aga Khan University Laboratory tested positive for the presence of uroporphyrins and Coproporphyrins. Before being diagnosed with VP, the patient had a hospital admission in Taj medical complex from 30-12-15 till 9-1-16 with complains of abdominal pain, loss of vision, and seizures for two days. At that time, a sample of the patient’s cerebrospinal fluid (CSF) was sent for analysis that came out normal. MRI brain with contrast was performed on 31-12-15 which showed evidence of cortical and sub-cortical areas of abnormal signal intensity in the bilateral parieto-occipital cortex and temporal cortex. Along with this there was increased meningeal enhancement within the basal cistern and overlying cortical sulci with associated patchy nodular enhancement in parieto-occipital, temporal and frontal region suggesting of meningo-encephalitis. During that hospital admission, the child was treated, perhaps incorrectly as viral encephalitis and discharged when stable.

When the child presented to NICH, he was a pre-pubertal male child of height 140 cm, weight 32 kg and vitals as follows: blood pressure, 140/100 mmHg (reference, 119/78 mmHg); pulse, 88 bpm; respiratory rate, 25 breaths/minute; and temperature, 98.6°F.

**Figure 1:**
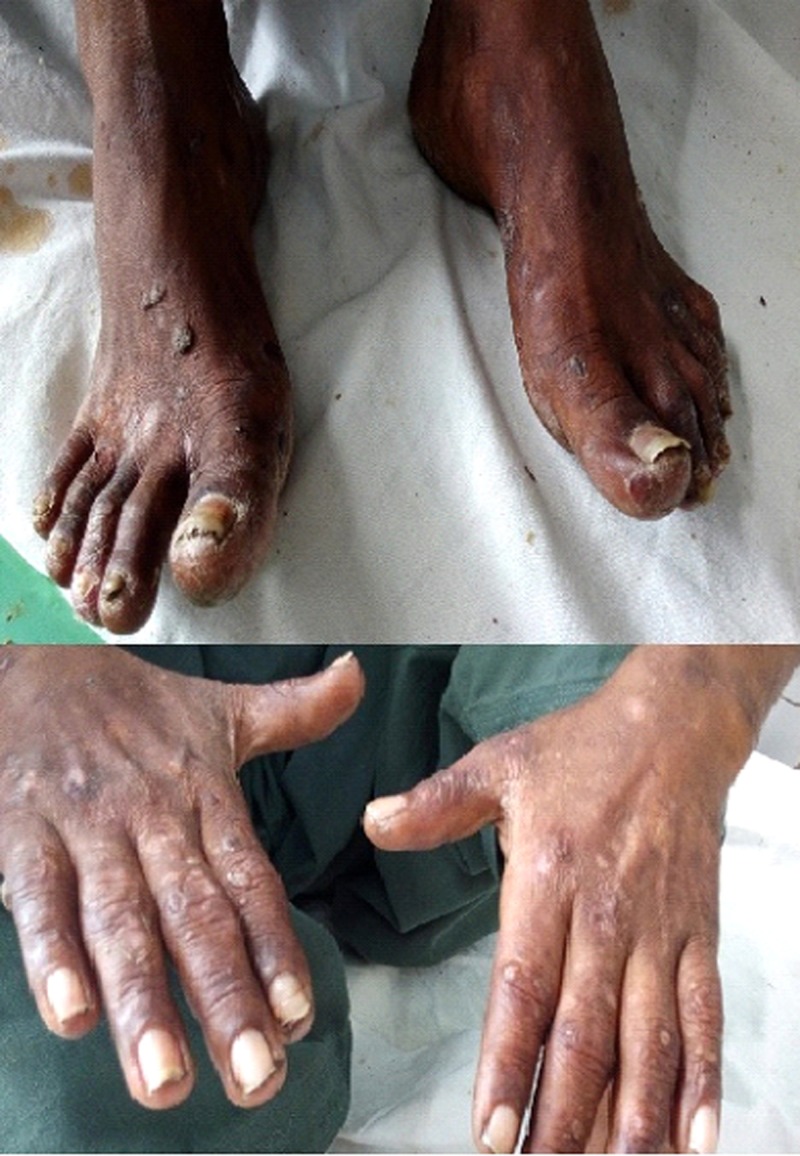
Skin lesions.

He also had multiple vesicles and bullae over the body in areas exposed to sunlight. The secondary skin changes due to the presence of these vesicular and bullous lesions included skin fragility with erosions from mild shearing trauma, hyper or hypo pigmentations of skin exposed to sunlight, melanosis and violaceous brown discoloration, milia, pseudo-scleroderma, atrophy and scarring of healed skin, alopecia, dystrophic calcification, and non-healing ulceration (Figure 1).

His neurological examination revealed a Glasgow coma scale (GCS) of 9/15 and bilaterally up-going plantars. There were no signs of meningeal irritation or cerebellar damage. Sensory system was intact and the back examination was unremarkable. Motor examination showed normal bulk in all four limbs, increased tone in all four limbs, power of 3/5 and hyperreflexia in all four limbs.

Fundoscopic examination showed a normal optic disc with dilated tortuous vessels. Rest of the systemic examination was unremarkable.

Consistent with these findings emergency department doctors kept hypertensive encephalopathy, infective encephalitis, and acute disseminated encephalomyelitis (ADEM) as provisional diagnosis. The blood pressure (BP) of the patient was monitored four-hourly as shown in Table 1.

**Table 1:** Four-hourly monitoring of blood pressure (Units: mm/Hg).

	1/12/2017	2/12/2017	3/12/2017	4/12/2017	5/12/2017	6/12/2017	7/12/2017
First reading	130/80	130/90	100/60	100/70	110/80	100/70	110/70
Second reading	140/100	120/90	120/70	120/90	110/70	100/70	110/70
Third reading	130/80	130/100	120/90	110/80	110/80	100/70	100/70
Fourth reading	130/90	110/90	120/80	110/70	110/70	100/70	100/70
Fifth reading	130/80	100/80	120/90	110/90	110/80	100/70	110/70

Investigations were then performed which included CSF analysis that showed a glucose of 58 mg/dL and protein of 20 mg/dL. Red and white blood cells were absent. The pre-lumbar puncture random blood sugar was 100 mg/dL. MRI brain was performed on 1-12-2017 which showed evidence of multifocal signal appearing hypo-intense on T1 and hyper-intense on T2 and fluid attenuation inversion recovery (FLAIR) image involving both cerebral and cerebellar hemispheres showing bilateral asymmetrical distributions. Signal abnormality was seen predominantly affecting the cortical grey matter with relative sparing of the white matter. Swollen gyri also showed restricted diffusion of T1 image. There was no pathological post-contrast enhancement seen. Rest of the brain parenchyma appeared normal.

Neurological opinion was taken and the neurologist suggested that the symptoms experienced by the patient seem to be a complication of porphyria. The neurologist further suggested that an MRI should be repeated to confirm the diagnosis which will show the disappearances of the abnormal signals present in the current MRI. Repeat MRI showing reversal of radiological findings is hallmark of PRES [2]. This was observed in the follow-up MRI done in our case after two weeks (13-12-17). The neurologist also advised to counsel the patient regarding the drugs, which may worsen his disease, that the patient should avoid in the future. A radiological opinion was also taken that stated that in view of the patient’s history the MRI findings were most likely due to PRES.

Before the diagnosis of PRES was made, the patient was initially treated on the lines of encephalitis in our hospital with injection Acyclovir 300 mg intravenous eight hourly, tablet Captopril 12.5 mg once orally, tablet Amlodipine 5 mg once orally and injection Methylprednisolone 650 mg once intravenously. While corticosteroids help in relieving vasogenic edema, no evidence is available for their efficacy in PRES [6]. As per our knowledge, till date no clinical trials have been done to describe comprehensively the management for PRES but rapid withdrawal of offending agent appears to speed up recovery and avoids complications; for example, aggressive blood pressure management, withdrawal of offending drug and delivery in case of Eclampsia [6, 8]. The factor that may have caused the exacerbation of this patient’s porphyria and development of PRES may be the increase in blood pressure. As we were successfully able to manage hypertension in our patient, the patient’s condition improved significantly on third post admission day; he regained consciousness and his vision improved.

**Figure 2:**
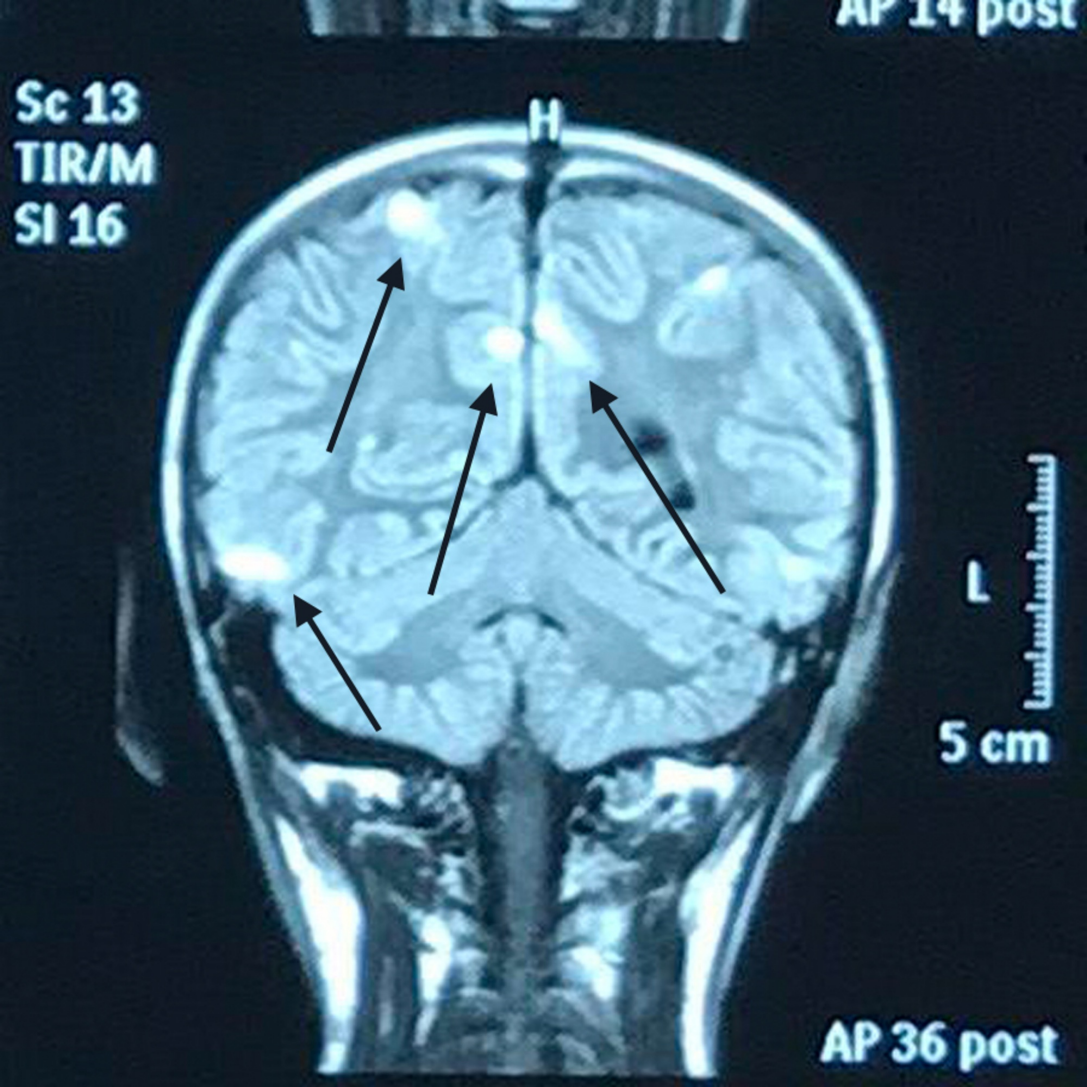
Follow-up magnetic resonance imaging (MRI) of the brain showing abnormal signal intensity areas in the parietal region appearing hyper-intense on fluid attenuation inversion recovery (FLAIR) image.

The follow-up re-demonstrated abnormal signal intensity areas in the frontal, posterior, parietal and occipital region in the cortical and sub-cortical areas bilaterally but more on the right side. They appeared hyper-intense on T2 and FLAIR image (Figure 2). Post contrast studies however showed no abnormal enhancement. On T1 image, restricted diffusion is seen only in the lesion within the frontal lobe on the right side. The scan of the remaining brain parenchyma appeared normal.

As the patient was a known case of variegate porphyria, the above findings were likely due to PRES. In contrast to the scan performed on 1-12-17 there was interval reduction in disease process. So a final diagnosis of variegate porphyria with PRES was made. Since the condition of the patient was improving, he was discharged on oral medications and a list of drugs to avoid. He was asked to follow up regularly on outpatient department basis.

## Discussion

VP is an acute porphyria and a cutaneous porphyria, an autosomal dominant hepatic porphyria due to deficiency of an enzyme called PPOX which is involved in the seventh step of heme synthesis. Mutation occurs in the gene encoding the mitochondrial enzyme PPOX. Most of the people who inherit this disease remain asymptomatic for life. This can be explained by the fact that although it is an autosomal dominant disease, the gene defect causing it has an incomplete penetrance and the disease remains in latent form [9]. This may be the reason that our case had no family history of the disease.

The major clinical manifestation of this disorder is the life threatening acute neurological attacks, typically occurring after puberty [10], however our case was a pre-pubertal male. The diagnosis of VP is suggested by a triad of symptoms of abdominal pain, neuropathy, and mental changes and confirmed by laboratory testing and finding a pathogenic heterozygous variant in PPOX [9,11]. Ninety percent of the patients present with autonomic changes like tachycardia and labile hypertension associated with abdominal pain, constipation, nausea and vomiting. While 10% present with central nervous system changes in the form of seizures, impaired consciousness, mental changes, and encephalopathy [12].

PRES is a clinico-radiological entity and a rare presenting feature of porphyria [3]. The brain typically shows focal regions of hemispheric edema on radiological scans with the parietal and temporal lobes most commonly affected, followed by frontal lobes, the inferior temporal-occipital junction and the cerebellum [13]. Commonly PRES evolves over a matter of hours. More than 70% of patients with PRES are hypertensive, though a significant proportion have normal or only mildly raised blood pressure [7,14].

The major clinical conditions associated with PRES are autoimmune diseases especially vascular autoimmune diseases, chronic and acute kidney disease, preeclampsia, eclampsia, infection/sepsis/shock, cancer chemotherapy, transplantation including bone marrow or stem cell transplantation, hypertension, hemolytic uremic syndrome, thrombotic thrombocytopenia purpura, drug toxicity and hyperammonia. These diseases happen to trigger the onset of PRES [4,8,15]. The trigger in our case was probably hypertension.

In our literature review we found no reported case of PRES as a complication of VP. Only two reported cases of AIP with PRES were found which were reported by Diosely et al. in 2016 and Zhao et al. in 2014 [2,16].

Due to its varied/nonspecific manifestations PRES is often misdiagnosed, as in our case where an initial provisional diagnosis of hypertensive encephalopathy, infective encephalitis and ADEM was made and the patient was treated with injection acyclovir. Similarly many of the cases of acute porphyria are also misdiagnosed and treated with precipitating drugs that further aggravates the clinical condition and can lead to complications such as PRES [11]. Once a diagnosis of porphyria is confirmed the treatment generally involves avoidance of aggravating factors that increase porphyrin levels and the downregulation of heme synthesis by avoiding fasting and early treatment of inter-current illness. In hypertension-associated and drug-induced PRES, the therapy of choice is withdrawal of offending agent, immediate control of blood pressure, anti-convulsant therapy, and temporary renal replacement therapy (hemodialysis/peritoneal dialysis) if required. In systemic lupus erythematosus (SLE)-related PRES, aggressive treatment with corticosteroids and cyclophosphamide is effective. In our case as hypertension was the offending factor, controlling blood pressure caused reversal of the condition and significant clinical improvement in just three days [4,17,18].

## Conclusions

The case illustrates that PRES, a rare neuro-radiographic abnormality, can present with variegate porphyria. This has never been reported before. The patient presented with symptoms of visual disturbances, altered mental status, and seizures which can be due to other conditions such as hypertensive encephalopathy, infective encephalitis and ADEM. However, these turned out not to be the cause of patient’s symptoms. It was the presence of a normal CSF detailed report and the changes in MRI brain that led to diagnosis of PRES. Awareness of diverse clinico-radiological presentation of acute PRES is necessary in order to prevent its misdiagnosis and treatment delay. PRES has a very good outcome with early treatment and dangerous outcome with delayed or missed diagnosis.
